# Chemical Composition, Antioxidant, Antibacterial, and Hemolytic Properties of Ylang-Ylang (*Cananga odorata*) Essential Oil: Potential Therapeutic Applications in Dermatology

**DOI:** 10.3390/ph17101376

**Published:** 2024-10-16

**Authors:** Soukaina Alaoui Mrani, Hind Zejli, Dounia Azzouni, Driss Fadili, Mohammed M. Alanazi, Said Omar Said Hassane, Rachid Sabbahi, Atul Kabra, Abdelfattah El Moussaoui, Belkheir Hammouti, Mustapha Taleb

**Affiliations:** 1 Laboratory of Engineering, Electrochemistry, Modelling and Environment, Faculty of Sciences, Sidi Mohamed Ben Abdellah University, Fez 30000, Morocco; 2 Laboratory of Chemical Physics, Materials and Environment, Faculty of Science and Technology, Moulay Ismaïl University of Meknes, Errachidia 52003, Morocco; 3 Department of Pharmaceutical Chemistry, College of Pharmacy, King Saud University, Riyadh 11451, Saudi Arabia; 4 Faculty of Sciences and Technology, University of Comoros, Moroni BP 2585, Comoros; 5 Research Team in Science and Technology, Higher School of Technology, University of Ibn Zohr, Laayoune 70000, Morocco; 6 University Institute of Pharma Sciences, Chandigarh University, Mohali 140307, India; 7 Plant Biotechnology Team, Faculty of Sciences, Abdelmalek Essaadi University, Tetouan 93002, Morocco; 8 Polytechnic School, Euro-Mediterranean University of Fez, Fez 30100, Morocco

**Keywords:** *Cananga odorata*, phytochemical composition, antioxidant activity, antibacterial activity, hemolytic analysis, molecular docking

## Abstract

**Background/Objectives:** This study investigates the chemical composition, antioxidant, antibacterial, and hemolytic properties of ylang-ylang (*Cananga odorata*) essential oil, with a focus on its potential therapeutic applications for dermatological diseases and the importance of transforming such bioactive properties into a stable, safe, and effective formulation. **Methods/Rsults:** Essential oils were extracted from flowers harvested in northern Grande Comore using hydro distillation at three different distillation times to examine the impact on yield and quality. Gas chromatographic analysis identified a complex mixture of compounds, including linalool, geranyl acetate, and benzyl benzoate. Antioxidant activity was assessed using DPPH, FRAP, TAC, and beta-carotene bleaching inhibition assays, revealing significant radical scavenging capabilities, with DPPH IC50 varying between 1.57 and 3.5 mg/mL. Antibacterial activity was tested against *Escherichia coli*, *Staphylococcus aureus*, *Bacillus subtilis*, and *Pseudomonas aeruginosa*, showing promising inhibition zones and minimum inhibitory concentrations. Hemolytic tests indicated varying degrees of red blood cell damage, emphasizing the need for careful concentration management in therapeutic applications. Molecular docking studies highlighted potential therapeutic targets for dermatological conditions, identifying high binding affinities for specific compounds against proteins involved in acne, eczema, and psoriasis. **Conclusions:** This comprehensive analysis underscores the potential of ylang-ylang essential oil (*YEOs*) as a natural alternative for antimicrobial treatments and dermatological applications, with its success dependent on optimized extraction methods and precise formulation to reduce cytotoxic effects. A formulation approach is crucial to ensure controlled release, improve bioavailability, and minimize skin irritation.

## 1. Introduction

The potential of essential oils as antimicrobial agents has been widely recognized, owing to their complex chemical compositions and historical use in traditional medicine [1,2,3]. Extracted from various plants, these oils contain compounds such as terpenes, phenolics, and other volatile molecules known for their biological activities [4,5]. Essential oils have proven effective against a broad spectrum of pathogens, including bacteria, fungi, viruses, and even some parasites. Their antimicrobial efficacy stems from mechanisms like disrupting cell membranes, interfering with cellular processes, and affecting microbial enzymes [6,7]. Researchers are increasingly exploring their applications in medicine, agriculture, and industry, highlighting their potential as natural alternatives to conventional antimicrobial agents [8,9,10].

Ylang-ylang (*Cananga odorata*), a tropical tree, is renowned for its fragrant essential oil used extensively in perfumery and aromatherapy. This oil, derived from the flowers of the ylang-ylang tree, contains active compounds (Linalool, Geraniol, Eugenol, Caryophyllene, Benzyl acetate, α-Farnesene, Benzyl Benzoate, β-Amyrin, Linalool, Germacrene D) which contribute to its aromatic qualities and biological activities, including antimicrobial effects [11,12]. Studies have shown that ylang-ylang essential oil exhibits antimicrobial activity against various microorganisms, including bacteria like *Staphylococcus aureus*, *Escherichia coli*, and *Pseudomonas aeruginosa*, as well as fungi such as *Candida albicans* and *Aspergillus niger* [13]. The antimicrobial properties of the oil are primarily due to its ability to disrupt microbial cell membranes, inhibit the synthesis of essential enzymes and proteins, and interfere with microbial biofilm formation. These characteristics make ylang-ylang essential oil a promising natural alternative to synthetic antimicrobials, with potential applications in treating skin infections, as a natural pesticide and fungicide in agriculture, and in the cosmetic and personal care industries for its fragrance and antimicrobial benefits [14,15].

The area of cultivation significantly impacts the antimicrobial activity of ylang-ylang essential oil due to various environmental and agricultural factors. Climate, including temperature, humidity, and rainfall, plays a crucial role in the growth and development of ylang-ylang trees, affecting the oil’s concentration and composition. Soil quality, encompassing type, pH, and nutrient content, influences the health of the plants and the quality of the oil produced. Additionally, the altitude at which ylang-ylang is grown can lead to variations in the concentration of active compounds, potentially impacting antimicrobial efficacy. The timing of harvest and extraction methods (e.g., steam distillation, cold pressing) further influence the chemical composition and antimicrobial activity of the oil [16]. Geographic origin is another critical factor, as different regions provide unique environmental conditions that result in variations in the oil’s chemical profile. For instance, ylang-ylang oil from Madagascar may differ significantly from oil produced in the Philippines, Indonesia, and the Comoros in terms of its major constituents and antimicrobial properties. This variability underscores the importance of considering the source and cultivation conditions when evaluating the efficacy of ylang-ylang essential oil for antimicrobial applications.

The aim of this study was to evaluate the properties and potential therapeutic applications of ylang-ylang essential oils (*YEOs*), focusing particularly on their antioxidant and antibacterial activities, as well as their hemolytic effects. The study also aimed to identify therapeutic targets for dermatological diseases via the molecular docking of molecules extracted from ylang-ylang oil.

## 2. Results and Discussion

### 2.1. Essential Oils’ Chemical Composition

The extraction of *YEOs* from *Cananga odorata* flowers resulted in three samples, namely Y1, Y2, and Y3 with the extraction times 6 h, 8 h, and 12 h and the oils color varied from golden to dark yellow, respectively. The obtained *YEOs* were then subjected to gas chromatography–mass spectrometry (GC–MS) analysis, and the results are presented in Table 1.

Felicia Ng et al. [17] have studied the difference in composition between ylang-ylang essential oil from the Comoros Islands and that from Madagascar. Their findings showed that oils from Madagascar are dominated by geranyl acetate, cis-α-farnesene, benzyl benzoate, humulene, and caryophyllene, whereas higher contents of cinnamyl acetate, benzyl salicylate, trans-farnesyl acetate, and α-farnesene were found in oils from Comoros.

The phytochemical analysis of *YEOs* conducted in this study identified the predominant compounds for the three samples. The main constituents were Geranyl acetate (5.42%, 4.18%, and 1.4%, respectively), Caryophyllene (5.27%, 1.17%, and 8.83%, respectively), Germacrene D (7.26%, 2.93%, and 15.30%, respectively), Benzyl acetate (2.82%, 5.02%, and 0.55%, respectively) and finally α-Farnesene (13.65%, 3.03%, and 24.80%, respectively). The predominant compounds that changed across samples were Benzyl Benzoate (10.52%) for Y1 and β-Amyrin (25.84%), Tetrapentacontane (15.72%), and Linalool (10.52%) for Y2 and δ-Cadinene (3.72% for Y1 and 6.00% for Y3).

### 2.2. Antioxidant Activities

The exploration of the antioxidant properties of *YEOs* (Table 2), extracted at different times, reveals significant differences in its ability to neutralize free radicals, as evidenced by the results of the DPPH and FRAP tests.

In the DPPH test, the essential oil Y2 stands out with notable efficacy, exhibiting an IC_50_ value of 1.57 ± 0.08 mg/mL, suggesting substantial antioxidant activity. These findings are supported by the FRAP test, where the oil extracted at the same time also demonstrates a low EC_50_ of 0.17 ± 0.04 mg/mL, indicating a strong ability to reduce ferricyanide iron.

These observations underscore the significant impact of extraction time on the specific antioxidant properties of ylang-ylang essential oil. The essential oil Y2 appears to promote the retention and/or formation of antioxidant compounds, resulting in increased activity in both the DPPH and FRAP tests.

However, despite time variations, all examined *YEOs* exhibit relatively similar levels of antioxidant activity in the beta-carotene bleaching test, with activity percentages ranging from 57 to 59%. This consistency suggests that different extraction times may have varying impacts on the specific antioxidant mechanisms targeted by the DPPH and FRAP tests, while maintaining overall similar activity.

**Figure 1:**
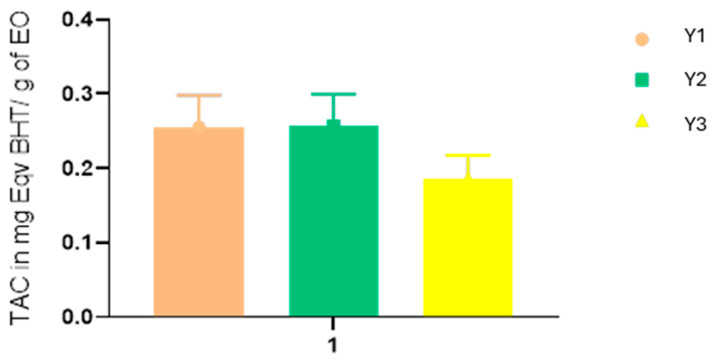
Total antioxidant capacity of *YEOs.*

Regarding the total antioxidant capacity of *YEOs*, the data in Figure 1 indicate that the extraction time significantly influences the antioxidant properties. The sample Y2 shows the highest antioxidant capacity, approximately 0.30 mg Eqv BHT/g, while Y1 and Y3 exhibit lower capacities at around 0.25 mg Eqv BHT/g and 0.15 mg Eqv BHT/g, respectively. These results align with the findings from DPPH and FRAP tests, demonstrating that the antioxidant activity changes with varying extraction times.

Furthermore, upon comparison with the existing literature, our results generally align with previous research, particularly concerning the DPPH and beta-carotene bleaching tests. For instance, Loucif et al. [18] reported DPPH values of 2.21 mg/mL, while Zejli et al. [19] found a relative antioxidant activity of 75% in the beta-carotene bleaching test. These findings suggest consistency and reliability in the antioxidant efficacy of *YEOs* across various studies.

### 2.3. Antibacterial Activity

Our investigation (Table 3) centered on evaluating the antibacterial activity of *YEOs* extracted at three distinct times against a panel of bacterial pathogens. Notably, the oil exhibited consistent inhibitory effects against *Staphylococcus aureus* and *Bacillus subtilis* across all extraction times, with mean inhibition diameters of 14.5 mm and minimum inhibitory concentrations (MICs) of 0.04 mg/mL. These results underscore the inherent antibacterial properties of ylang-ylang essential oil, which appear to be independent of the extraction time, suggesting its potential as a reliable antimicrobial agent against Gram-positive bacteria.

However, the effectiveness of *YEOs* against *E. coli* displayed variability across the different extraction times. Inhibition diameters ranged from “Not Found” (NF) to approximately 17.11 mm, with corresponding MICs of 0.01 to 0.02 mg/mL. This variability may stem from differences in the chemical composition of the oil at varying extraction times, influencing its interaction with *E. coli*. Furthermore, *P. aeruginosa* demonstrated resistance to *YEOs* across all extraction times, indicating a need for alternative approaches to address infections caused by this resilient pathogen.

In comparison to the positive control kanamycin, *YEOs* displayed promising antibacterial activity, particularly against Gram-positive bacteria. These findings underscore the significant antibacterial potential of *YEOs* across different extraction times. Aligning with the existing literature, our results contribute to the growing body of evidence supporting the remarkable antibacterial activity inherent in *YEOs*. Numerous studies have underscored its invaluable antibacterial properties, highlighting its potential as a natural alternative or complement to conventional antibiotics [20,21].

### 2.4. Hemolytic Test

**Figure 2:**
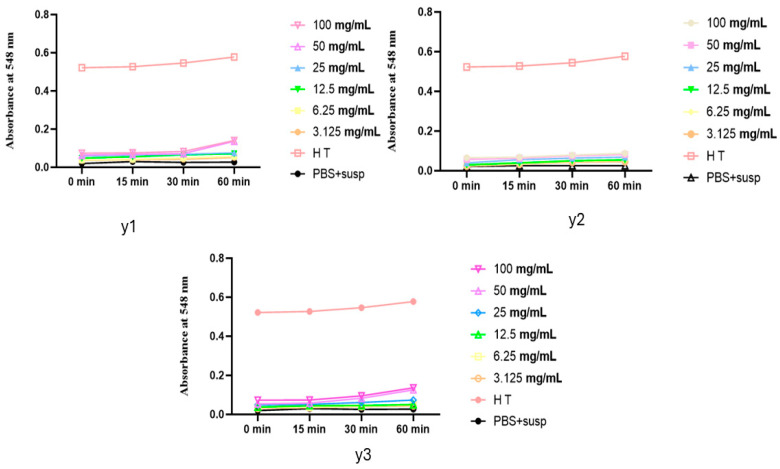
Absorbance changes over time during incubation (37 °C) of erythrocyte suspensions with varying concentrations of *YEOs*, PBS + susp: red blood cell suspension from rat blood was incubated in a phosphate-buffered saline (PBS) solution at pH 7.4.

The hemolytic test results, shown in the three graphs, demonstrate the absorbance changes over time during the incubation (37 °C) of erythrocyte suspensions with varying concentrations of *YEOs* (Figure 2).

In all three graphs, the absorbance levels of the oil-treated samples are lower than the positive control (HT), indicating no significant hemolysis.

Graph Y1 and Graph Y3 show slight increases in absorbance at higher concentrations (100 mg/mL and 50 mg/mL). However, these values remain below 0.2, far below the 1.3 absorbance observed in total hemolysis. This suggests that even at higher concentrations, *YEOs* do not cause significant hemolysis.

Graph Y2 shows absorbance levels almost identical to the negative control (PBS + susp) for all concentrations, indicating no hemolytic activity. This uniformity across all concentrations implies that the essential oil does not induce hemolysis.

Our results align with findings in the literature, which report the use of various formulations containing ylang-ylang without adverse effects or damage [19,22]. The consistency of these findings with our own results further supports the safety profile of *YEOs*. The absence of hemolytic activity at all tested concentrations demonstrates that *YEOs* can be safely used without causing harm to erythrocytes.

### 2.5. Identification of Therapeutic Targets for Dermatological Diseases via Molecular Docking

In this study, we focused on the dermatological properties of the molecules extracted from oil. Dermatological diseases, also known as skin diseases, cover an extensive range of conditions that affect the skin, hair, and nails. These conditions vary greatly in terms of severity, symptoms, and causes. They affect people regardless of their region, culture, or age, impacting anywhere between 30% and 70% of the population around the world [23].

Table 4 presents the molecular properties and rule violations for a set of extracted molecules. The results indicate that Molecules 1 and 2 are fully compliant with Lipinski’s, Ghose’s, and Veber’s rules, while Molecules 3 to 9 exhibit one or more violations of these rules. Specifically, Molecules 5 through 9 violate Lipinski’s rule due to high logP values, and Molecule 8 also exceeds the recommended molecular weight. Consequently, we split the studied molecules into two sets: those compliant with Lipinski’s rules and those that are non-compliant.

**Figure 3:**
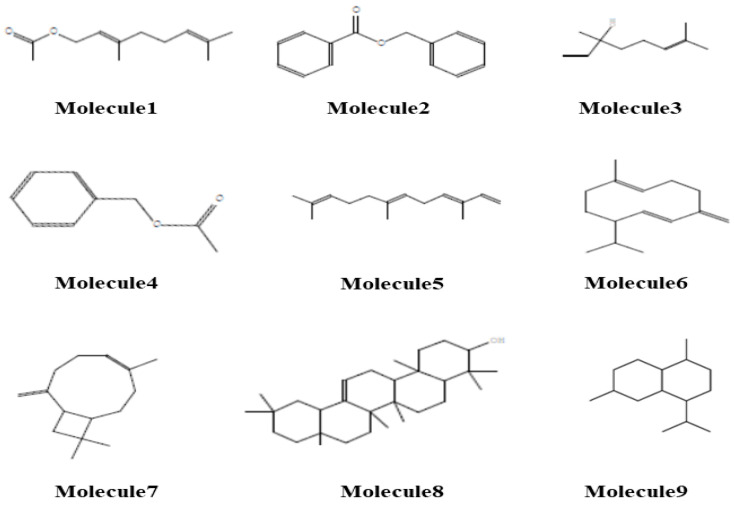
Molecular structures of target molecules.

The first set, comprising Molecules 1 to 4, exhibits high gastrointestinal (GI) absorption and strong blood–brain barrier (BBB) penetration, as illustrated in Figure 3. These molecules are not substrates for P-glycoprotein (Pgp) and do not inhibit major cytochrome P450 enzymes (CYP1A2, CYP2C19, CYP2C9, CYP2D6, and CYP3A4), with the exception of Molecules 2 and 4, which inhibit CYP1A2 and CYP2C19, respectively, indicating that these compounds may have lower levels of metabolism and potentially lead to unwanted effects.

In contrast, the second set, comprising Molecules 5 to 9, exhibits low gastrointestinal (GI) absorption and a reduced ability to penetrate the blood–brain barrier (BBB), as depicted in Figure 3. These molecules are also not Pgp substrates; however, Molecules 5, 6, 7, and 9 inhibit various cytochrome P450 enzymes. This comprehensive analysis highlights the distinction between molecules that meet standard drug-likeness criteria and those that do not, thereby guiding further optimization and development efforts.

The treatment of acne vulgaris is based on the inhibition of 5α-reductase to reduce sebum production, or inhibiting acnes lipase, which aggravates acne through inflammation and the breakdown of sebum [24,25]. For eczema, inhibitors of phosphodiesterase 4 (PDE4) can reduce inflammation by increasing cyclic adenosine monophosphate (cAMP) levels in immune cells. Blocking interleukin signaling (IL-4 and IL-13) can also reduce the inflammatory response associated with eczema [26,27].

Inflammatory cytokines such as Interleukin-17 (IL-17), Interleukin-23 (IL-23), and Tumor Necrosis Factor-alpha (TNF-α) play a crucial role in the inflammatory response caused by the psoriasis, so targeting them could manage this skin condition [28,29]. Additionally, targeting Cathelicidin (LL-37) and Transient Receptor Potential (TRP) channels can reduce the inflammatory response and other symptoms, such as skin redness and pain, caused by rosacea [30].

For vitiligo, inhibiting Janus kinase (JAK) blocks IFN-γ signaling, which inhibits melanocyte proliferation and interferes with re-pigmentation processes [31]. Targeting the BRAFV600 mutation in metastatic melanoma with BRAF/MEK inhibitors can inhibit cancer cell growth [32].

**Figure 4:**
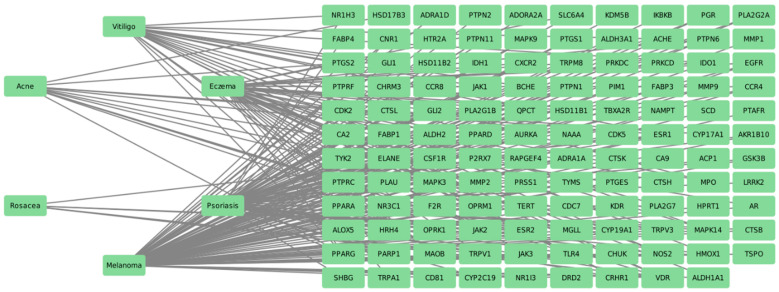
The interaction pharmacology network for the treatment of the selected dermatological diseases.

By using keywords such as “Acne”, “Eczema”, “Psoriasis”, “Rosacea”, “Vitiligo”, and “Melanoma” on DisGeNET [33], we identified potential therapeutic targets for these diseases. Figure 4 illustrates the interaction pharmacology network for the treatment of the selected dermatological diseases. The network includes 125 nodes (encompassing six diseases) and 234 edges. This network demonstrates that the treatment of each disease can target multiple genes, and targeting a single gene can be relevant to multiple diseases. This highlights the complex and interconnected nature of the pharmacological interactions among these diseases and their treatment targets.

To identify relevant therapeutic targets that are both biologically significant to the diseases and potentially modulated by the extracted molecules, we examined the intersection between the targets of the selected molecules and disease-specific targets. This approach ensures that the selected proteins for docking studies are biologically relevant to the diseases and have the potential to interact with the extracted molecules. Table 5 summarizes the key target genes modulated by the selected molecules for these pathologies.

## 3. Material and Methods

### 3.1. Plant Material

The *Cananga odorata* yellow flowers were harvested in northern Grande Comore (Mitsamihouli) during May and June 2021, early in the morning to ensure optimal volatile compound concentration. A botanical identification was performed, and a voucher code was assigned to this plant: AND45-HKM. The extraction was carried out using hydro distillation at three different extraction times to assess the impact on yield and quality. The first phase was conducted after 6 h, allowing for the extraction of the most volatile compounds without thermal degradation. The second phase, after 8 h, facilitated a more comprehensive extraction while minimizing the degradation of heat-sensitive compounds. The final phase was carried out after 12 h, aiming to maximize the overall yield by extracting the least volatile compounds, despite the potential degradation of certain thermolabile components.

### 3.2. Phytochemical Analysis

Gas chromatographic analysis was performed using a Shimadzu GCMS-TQ8040 NX system with an apolar capillary column (RTxi-5 Sil MS, 30.00 m length, 0.250 mm inner diameter, 0.250 μm film thickness). The essential oil was diluted in hexane with a dilution ratio of 10:100, and the volume of sample injected was 1 mL using the fractional injection technique. The temperature program started at 50 °C for 2 min, increased to 260 °C for 10 min, and then ramped up at 5 °C/min until it reached 280 °C. Nitrogen was used as the carrier gas at a flow rate of 1 mL/min. The injector and detector temperatures were set at 250 °C and 280 °C, respectively. The ion source temperature was set at 200 °C, and the interface line temperature was 280 °C, and the scan mass range was *m*/*z* 40–650. Volatile compounds in the essential oil were identified using NIST version 2019.

### 3.3. Antioxidant Activity

The antioxidant potential of *YEOs* was evaluated using four distinct in vitro assays:

*a.*  2,2-diphenylpicrylhydrazyl (DPPH) Method

In this procedure, 100 µL of each extract solution was combined with 750 µL of a methanolic DPPH solution (0.004%). Following a 30 min incubation at room temperature, the absorbance was measured at 517 nm [34]. The percentage of DPPH inhibition was determined employing the formula:

(1) $$PI\left(\%\right)=(\frac{A0-A}{A0})\times 100$$

where:

PI is Percentage of inhibition;

A0 is Absorbance of the DPPH of negative control;

A is Absorbance of DPPH of the sample;

IC_50_ values were obtained from the inhibition percentage graph against extract concentration.

*b.*  Ferric Reducing Antioxidant Power (FRAP) Test

To perform the FRAP assay, a solution was prepared by combining 500 µL of potassium ferricyanide (1%), 500 µL of phosphate buffer (0.2 M, pH 6.6), and 100 µL of various sample concentrations dispersed in methanol. The mixture was incubated at 50 °C for 20 min. After incubation, 500 µL of 10% aqueous TCA solution, 500 µL of distilled water, and 100 µL of 0.1% FeCl_3_ were added. Absorbance was measured at 700 nm, and the results were expressed as the 50% effective concentration (EC_50_) [35].

*c.*  Total Antioxidant Capacity (TAC) Test

In this assay, 25 µL of each sample was combined with a reagent solution composed of 28 mM sodium phosphate, 4 mM ammonium molybdate, and 0.6 M sulfuric acid. The mixture was heated at 95 °C for 90 min. After incubation, the absorbance was measured at 695 nm using a spectrophotometer. The total antioxidant capacity was determined by comparing the absorbance to a standard curve of ascorbic acid and expressed as micrograms of BHT equivalent per gram of sample (mg eqv BHT/g sample) [36].

*d.*  Beta-Carotene Bleaching Inhibition Assay

Based on the method described by Ozsoy et al. (2008) [37], this assay was employed to evaluate the effectiveness of each sample in inhibiting the bleaching of beta-carotene in a beta-carotene/linoleic acid system. Absorbance was recorded after 120 min for both the negative control (AE) and the positive control (APC). The percentage of antioxidant activity was then calculated using the formula:

(2) $$AA\;(\%)=\left(\frac{A_{E}}{A_{PC}}\right)\times 100$$

### 3.4. Antibacterial Activity

The antibacterial efficacy was initially evaluated through qualitative analysis employing the disk diffusion method [38], to identify potent extracts. The three samples underwent testing against *Escherichia coli* (ATCC 25922), *Staphylococcus aureus* (ATCC 29213), *Bacillus subtilis* (ATCC 6633), and *Pseudomonas aeruginosa* (ATCC 27853). Subsequently, the three essential oils were subjected to microdilution on 96-well microplates, following the protocol outlined by [39], to determine the minimum inhibitory concentration.

### 3.5. Hemolytic Test

In our study, we carried out an in vitro toxicity assay to investigate the hemolytic effects of three *YEOs*, extracted at different times, on red blood cells (RBCs). This analysis is critical given the frequent use of ylang-ylang in cream formulations. The procedure began by exposing RBCs to the essential oils at various concentrations, followed by incubation at 37 °C. After incubation, the mixture was subjected to centrifugation to separate the supernatant, which was then analyzed for absorbance at 540 nm. By comparing the absorbance readings from the RBCs treated with essential oils to those from established positive and negative controls, we calculated the percentage of hemolysis, which reflects RBC damage. This evaluation was conducted using a spectrophotometer at 548 nm. Hemolysis induced by the extracts was measured relative to control hemolysis [40,41].

### 3.6. Molecular Docking

In this study, the molecules extracted from *YEOs* were evaluated for their dermatological properties. Potential therapeutic targets were identified using the DisGeNET database with specific keywords for dermatological diseases such as acne, eczema, and psoriasis. Nine target proteins were selected, and their structures obtained from the RCSB protein database. Ligands were optimized using density functional theory (DFT) calculations, and PDB files were converted to PDBQT using AutoDock Tools. Docking was performed with AutoVina and results were visualized with Biovia Visualization Tools.

### 3.7. Statistical Analysis

Data analysis consisted of calculating the means of the three replicate analyses and presenting the results as mean ± standard deviation (SD). Statistical analysis was undertaken using IBM SPSS Statistics version 20.0. Fisher’s smallest significant difference (LSD) test and one-way analysis of variance (ANOVA) were used to determine statistical significance between the different groups, with significance set at *p* ≤ 0.05

## 4. Conclusions

The study concludes that ylang-ylang essential oils (*YEOs*) possess significant antioxidant and antibacterial activities, making it a promising natural alternative or complement to conventional antibiotics. The three oils, especially Y2, have demonstrated substantial DPPH inhibition and high total antioxidant capacity in various in vitro assays. Antibacterial tests also revealed its efficacy against several bacterial strains. Importantly, hemolytic tests showed that ylang-ylang essential oil does not cause significant damage to red blood cells, indicating that it is safe for topical use.

In addition, molecular docking studies have identified several potential therapeutic targets for dermatological diseases suggesting a promising therapeutic route for conditions such as acne, eczema, and psoriasis. This approach not only maximizes the therapeutic benefits of YEO, but also opens up new paths for treating skin infections and inflammatory conditions more effectively than applications of unrefined essential oils.

These findings highlight the potential of incorporating ylang-ylang essential oil into cosmetic and pharmaceutical formulations to prevent skin infection and inflammation, thanks to its antioxidant and antibacterial properties. Its safety profile means it can be incorporated into creams, lotions, and gels, opening up new possibilities for treating common skin conditions and advancing dermatological treatments.

## Figures and Tables

**Table 1:** Phytochemical constituents of ylang-ylang essential oil Y1, Y2, and Y3.

Name	Chemical Formula	Retention Index	Area %		
			Y1	Y2	Y3
Hexanal	C_6_H_12_O	800	-	1.36	-
2,4-Dimethylheptane	C_9_H_20_	1022	-	0.91	-
3-Methoxy-toluene	C_8_H_10_O	1031	1.73	4.78	-
Benzoic acid, methyl ester	C_8_H_8_O_2_	1079	1.46	3.76	-
Linalool	C_10_H_18_O	1095	4.19	10.52	-
Benzyl acetate	C_9_H_10_O_2_	1160	-	-	0.44
Benzyl acetate	C_9_H_10_O_2_	1165	2.82	5.02	0.55
Dodecane, 2,6,11-trimethyl	C_15_H_32_	1200	0.97	5.41	1.47
Nerol	C_10_H_18_O	1228	0.29	-	-
Cinnamyl alcohol	C_9_H_10_O	1259	1.96	-	-
Elemene	C_15_H_24_	1340	0.47	-	-
α-Copaene	C_15_H_24_	1377	1.06	-	1.90
Farnesan	C_15_H_32_	1381	-	2.19	1.15
Geranyl acetate	C_12_H_20_O_2_	1384	5.24	4.18	1.40
β-Funebrene	C_15_H_24_	1416	-	-	0.86
β-Caryophyllene	C_15_H_24_	1418	5.27	1.17	8.83
α-Guaiene	C_15_H_24_	1440	-	-	0.73
Phenol acetate	C_10_H_12_O_4_	1445	-	2.93	0.82
β-Farnesene	C_15_H_24_	1448	0.78	-	-
Muurola-4(14),5-diene	C_15_H_24_	1470	0.36	-	0.63
α-Caryophyllene	C_15_H_24_	1478	1.74	-	-
Germacrene D	C_15_H_24_	1490	7.26	2.93	15.30
α-Muurolene	C_15_H_24_	1503	0.25	-	0.43
γ-Muurolene	C_15_H_24_	1504	0.85	-	-
α-Muurolene	C_15_H_24_	1507	1.11	-	2.09
α-Farnesene	C_15_H_24_	1510	13.65	3.03	24.80
α-Amorphene	C_15_H_24_	1513	1.13	-	0.47
γ-Cadinene	C_15_H_24_	1517	1.66	-	6.34
δ-Cadinene	C_15_H_24_	1525	3.72	-	6.00
Zonarene	C_14_H_22_	1528	0.25	-	-
Copaen-11-ol	C_15_H_24_O	1544	0.62	-	1.17
Junenol	C_15_H_26_O	1618	0.48	-	0.47
α-Muurolol	C_15_H_26_O	1648	7.73	-	5.45
Farnesol	C_15_H_26_O	1686	0.65	-	-
Benzyl Benzoate	C_14_H_12_O_2_	1766	10.52	3.16	4.54
Octadecane	C_18_H_38_	1800	0.95	-	-
Farnesyl acetate (2Z,6E)	C_17_H_28_O_2_	1823	2.90	-	3.15
Benzyl salicylate	C_14_H_12_O_3_	1857	4.70	-	1.18
β-Amyrin	C_30_H_50_O	1980	-	25.84	-
Eicosane	C_20_H_42_	2000	0.80	5.97	2.57
Dotriacontane	C_32_H_66_	3204	2.08	15.72	3.06
Betunal	C_30_H_48_O_3_	3628	4.58	-	-
**TOTAL**			94.23	98.88	98.39

**Table 2:** Assessment of antioxidant properties of *YEOs*.

	Y1	Y2	Y3	BHT	Quercetin
**DPPH (IC_50_ mg/mL)**	3.5 ± 0.03	1.57 ± 0.08	1.91 ± 0.04	0.11 ± 0.001	-
**FRAP (EC_50_ mg/mL)**	0.21 ± 0.01	0.17 ± 0.04	0.19 ± 0.01	-	0.03 ± 0.004
**Relative antioxidant activity in %**	56.67%	58.67%	57.32%	100%	-

**Table 3:** Evaluation of antibacterial activity of *YEOs.*

	*E. coli*		*S. aureus*		*B. subtilis*		*P. aeruginosa*	
	ID (mm)	MIC mg/mL	ID (mm)	MIC mg/mL	ID (mm)	MIC mg/mL	ID (mm)	MIC mg/mL
**Y1**	NF	NF	14.5_±0.45_	0.04	11.00_±1.00_	0.04	NF	NF
**Y2**	17.11_±0.00_	0.02	12.5_±1.11_	0.01	18.05_±1.25_	0.02	NF	NF
**Y3**	NF	NF	14.5_±1.00_	0.04	14.00_±0.5_	0.04	NF	NF
**Kanamycin**	19.3_±1.56_	0.002	21.4_±1.2_	0.016	19.3_±1.5_	0.004	17.00_±0.00_	0.004

**Table 4:** Calculated descriptors relevant to orally bioavailable drug-like chemical space for the extracted molecules.

Molecule	Formula	MW	RT	HA	HD	MR	TPSA	MlogP	Lipinski	Ghose	Veber
									Violations		
Molecule 1 (*Geranyl acetate*)	C_12_H_20_O_2_	196.29	6	2	0	60.13	26.3	2.95	0	0	0
Molecule 2 (*benzyl benzoate*)	C_14_H_12_O_2_	212.24	4	2	0	62.21	26.3	3.41	0	0	0
Molecule 3 (*Linalool*)	C_10_H_18_O	154.25	4	1	1	50.44	20.23	2.59	0	1	0
Molecule 4 (*Benzyl acetate*)	C_9_H_10_O_2_	150.17	3	2	0	42.31	26.3	1.98	0	1	0
Molecule 5 (*α-Farnesene*)	C_15_H_24_	204.35	6	0	0	72.32	0	4.84	1	0	0
Molecule 6 (*Germacrene-D*)	C_15_H_24_	204.35	1	0	0	70.68	0	4.53	1	0	0
Molecule 7 (Caryophylene)	C_15_H_24_	204.35	0	0	0	68.78	0	4.63	1	0	0
Molecule 8 (*β-Amyrin*)	C_30_H_50_O	426.72	0	1	1	134.88	20.23	6.92	1	3	0
Molecule 9 (*δ-Cadinene*)	C_15_H_24_	204.35	1	0	0	69.04	0	4.63	1	0	0

**Table 5:** Gene targeted by more than two molecules.

Disease	Gene	Molecule	PDB ID
**Acne**	AR	Molecule 3, Molecule 6, Molecule 8	1E3G
	CYP17A1	Molecule 1, Molecule 2, Molecule 8	1E6A
	CYP19A1	Molecule 4, Molecule 6, Molecule 8	3S7R
**Eczema**	CA2	Molecule 1, Molecule 2, Molecule 3, Molecule 4	1CA2
	JAK2	Molecule 1, Molecule 3, Molecule 4	3KCK
	JAK3	Molecule 1, Molecule 3, Molecule 4	4Z16
	PPARA	Molecule 6, Molecule 8, Molecule 9	1K7L
**Psoriasis**	CA2	Molecule 1, Molecule 2, Molecule 3, Molecule 4	1CA2
	ESR2	Molecule 2, Molecule 6, Molecule 8	3OLS
	JAK2	Molecule 1, Molecule 3, Molecule 4	3KCK
	PPARA	Molecule 6, Molecule 8, Molecule 9	1K7L
**Rosacea**	CYP19A1	Molecule 4, Molecule 6, Molecule 8	3S7R
**Vitiligo**	PRSS1	Molecule 1, Molecule 2, Molecule 4	3R43
**Melanoma**	AR	Molecule 3, Molecule 6, Molecule 8	1E3G
	CA2	Molecule 1, Molecule 2, Molecule 3, Molecule 4	1CA2
	CYP19A1	Molecule 4, Molecule 6, Molecule 8	3S7R
	ESR2	Molecule 2, Molecule 6, Molecule 8	3OLS
	JAK2	Molecule 1, Molecule 3, Molecule 4	3KCK
	JAK3	Molecule 1, Molecule 3, Molecule 4	4Z16
	PPARA	Molecule 6, Molecule 8, Molecule 9	1K7L
	PRSS1	Molecule 1, Molecule 2, Molecule 4	3R43

## Data Availability

The original contributions presented in the study are included in the article.
